# Investigating the Effects of Frontotemporal Dementia‐Linked Genetic Mutations on Inequity Aversion Prior to Clinical Onset

**DOI:** 10.1002/alz70857_102530

**Published:** 2025-12-24

**Authors:** Chenyu Wang

**Affiliations:** ^1^ UCSF, san francisco, CA, USA

## Abstract

**Background:**

Frontotemporal dementia (FTD) is a neurodegenerative disorder associated with behavioral and cognitive impairments. This study hypothesized that individuals carrying FTD‐linked genetic mutations exhibit altered inequity aversion compared to non‐carriers, even before clinical symptoms emerge. To test this, 102 participants were recruited, each completing 20 rounds of a modified Dictator Game to measure inequity aversion through monetary allocation tasks with varying token values.

**Method:**

The sensitivity and consistency of inequity aversion were assessed using behavioral models. Sensitivity was analyzed using a stochastic utility‐based model with parameters α∖alpha and β∖beta quantifying aversion to advantageous and disadvantageous inequity, respectively. Probabilities of choices were modeled with a SoftMax function, and Maximum Likelihood Estimation with Bootstrap was applied to estimate parameters. Consistency was evaluated by the frequency of violations of the General Axiom of Revealed Preferences (GARP), reflecting choice inconsistency. Statistical comparisons between genetic mutation carriers and non‐carriers were conducted using linear mixed‐effects models, Kolmogorov‐Smirnov tests, Wilcoxon Rank‐Sum Tests, and Fisher's exact tests.

**Result:**

There were no significant differences between genetic carriers and non‐carriers in either the sensitivity (*p* > 0.05) or consistency (*p* > 0.05) of inequity aversion. Visualizations and statistical tests consistently showed overlapping distributions of behavioral measures across groups.

**Conclusion:**

This study found no significant differences in inequity aversion between mutation carriers and non‐carriers before the onset of FTD symptoms. These findings suggest that behavioral markers of cognitive changes in FTD may emerge only within a narrow temporal window before disease onset, presenting challenges for early detection in asymptomatic individuals. Future studies should explore complementary approaches to identify prodromal biomarkers of FTD.

